# 154. Circulation of Rhinovirus/Enterovirus Respiratory Infections in Children During 2020-21 in the United States

**DOI:** 10.1093/ofid/ofab466.154

**Published:** 2021-12-04

**Authors:** Danielle A Rankin, Andrew Speaker, Ariana Perez, Zaid Haddadin, Varvara Probst, Jennifer E Schuster, Anna L Blozinski, Herdi Kurnia Rahman, Laura S Stewart, Brian Rha, Marian G Michaels, John V Williams, Julie A Boom, Leila C Sahni, Mary Allen Staat, Elizabeth P Schlaudecker, Monica McNeal, Rangaraj Selvarangan, Christopher J Harrison, Geoffrey A Weinberg, Peter G Szilagyi, Janet A Englund, Eileen J Klein, Meredith McMorrow, Manish Patel, James Chappell, Claire Midgley, Natasha B Halasa, Natasha B Halasa

**Affiliations:** 1 Vanderbilt University Medical Center; Division of Pediatric Infectious Diseases, Nashville, TN; 2 Vanderbilt University Medical Center, Nashville, TN; 3 Centers for Disease Control and Prevention, Atlanta, Georgia; 4 University of Florida, Jacksonville, Jacksonville, Florida; 5 Children’s Mercy Hospital, Kansas City, MO; 6 Children’s Hospital of Pittsburgh of UPMC, Pittsburgh, PA; 7 University of Pittsburgh, Pittsburgh, Pennsylvania; 8 Baylor College of Medicine, Houston, TX; 9 Texas Children’s Hospital, Houston, Texas; 10 Cincinnati Children’s Hospital Medical Center, Cincinnati, OH; 11 Cincinnati Children’s Hospital Medical Center, University of Cincinnati College of Medicine, Cincinnati, OH; 12 University of Rochester, Rochester, New York; 13 University of California, Los Angeles, Los Angeles, California; 14 Seattle Children’s Hospital/Univ. of Washington, Seattle, Washington; 15 Seattle Children’s Hospital, Seattle, Washington; 16 CDC, Georgia

## Abstract

**Background:**

Sharp declines in influenza and respiratory syncytial virus (RSV) circulation across the U.S. have been described during the pandemic in temporal association with community mitigation for control of severe acute respiratory syndrome coronavirus 2 (SARS-CoV-2). We aimed to determine relative frequencies of rhinovirus/enterovirus (RV/EV) and other respiratory viruses in children presenting to emergency departments or hospitalized with acute respiratory illness (ARI) prior to and during the COVID-19 pandemic.

**Methods:**

We conducted a multi-center active prospective ARI surveillance study in children as part of the New Vaccine Surveillance Network (NVSN) from December 2016 through January 2021. Molecular testing for RV/EV, RSV, influenza, and other respiratory viruses [i.e., human metapneumovirus, parainfluenza virus (Types 1-4), and adenovirus] were performed on specimens collected from children enrolled children. Cumulative percent positivity of each virus type during March 2020–January 2021 was compared from March-January in the prior seasons (2017-2018, 2018-2019, 2019-2020) using Pearson’s chi-squared. Data are provisional.

**Results:**

Among 69,403 eligible children, 37,676 (54%) were enrolled and tested for respiratory viruses. The number of both eligible and enrolled children declined in early 2020 (Figure 1), but 4,691 children (52% of eligible) were enrolled and tested during March 2020-January 2021. From March 2020-January 2021, the overall percentage of enrolled children with respiratory testing who had detectable RV/EV was similar compared to the same time period in 2017-2018 and 2019-2020 (Figure 1, Table 1). In contrast, the percent positivity of RSV, influenza, and other respiratory viruses combined declined compared to prior years, (p< 0.001, Figure 1, Table 1).

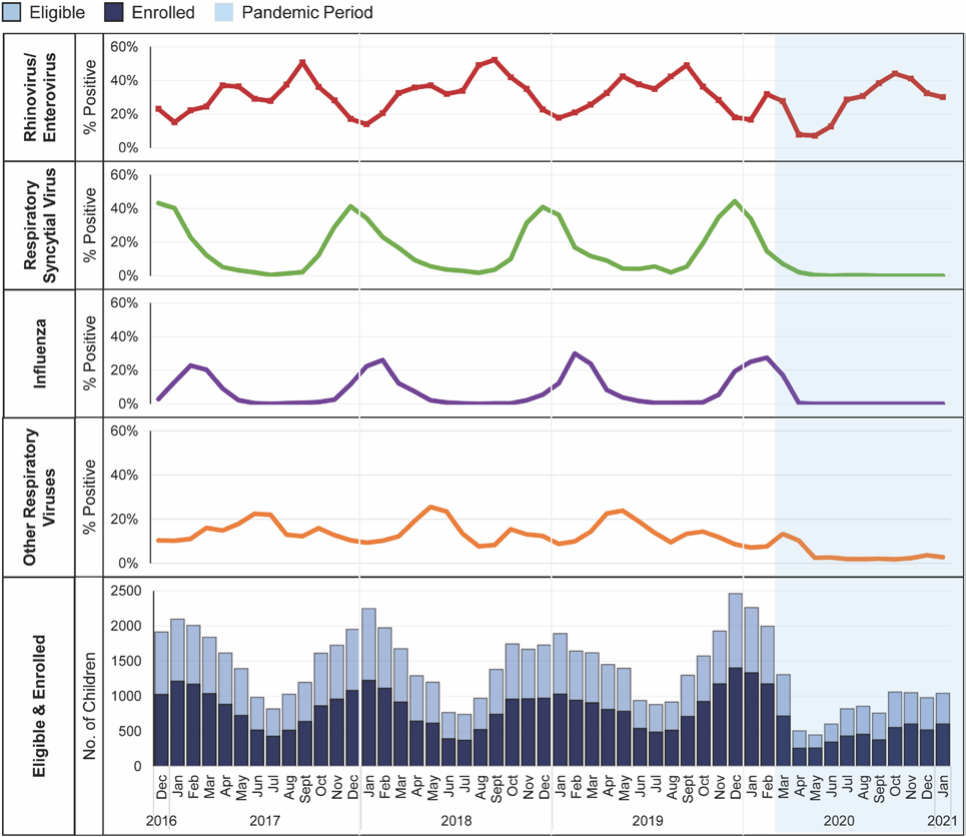

Figure 1. Percentage of Viral Detection Among Enrolled Children Who Received Respiratory Testing, New Vaccine Surveillance Network (NVSN), United States, December 2016 – January 2021

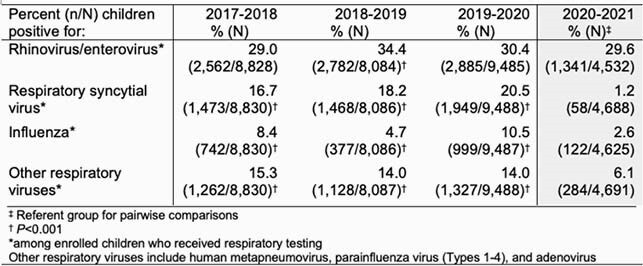

Table 1. Percent of Respiratory Viruses Circulating in March 2020– January 2021, compared to March-January in Prior Years, New Vaccine Surveillance Network (NVSN), United States, March 2017 – January 2021

**Conclusion:**

During 2020, RV/EV continued to circulate among children receiving care for ARI despite abrupt declines in other respiratory viruses within this population. These findings warrant further studies to understand virologic, behavioral, biological, and/or environmental factors associated with this continued RV/EV circulation.

**Disclosures:**

**Jennifer E. Schuster, MD**, Merck, Sharpe, and Dohme (Individual(s) Involved: Self): Grant/Research Support **Marian G. Michaels, MD, MPH**, **Viracor** (Grant/Research Support, performs assay for research study no financial support) **John V. Williams, MD**, **GlaxoSmithKline** (Advisor or Review Panel member, Independent Data Monitoring Committee)**Quidel** (Advisor or Review Panel member, Scientific Advisory Board) **Elizabeth P. Schlaudecker, MD, MPH**, **Pfizer** (Grant/Research Support)**Sanofi Pasteur** (Advisor or Review Panel member) **Christopher J. Harrison, MD**, **GSK** (Grant/Research Support)**Merck** (Grant/Research Support)**Pfizer** (Grant/Research Support, Scientific Research Study Investigator, Research Grant or Support) **Janet A. Englund, MD**, **AstraZeneca** (Consultant, Grant/Research Support)**GlaxoSmithKline** (Research Grant or Support)**Meissa Vaccines** (Consultant)**Pfizer** (Research Grant or Support)**Sanofi Pasteur** (Consultant)**Teva Pharmaceuticals** (Consultant) **Claire Midgley, PhD**, Nothing to disclose **Natasha B. Halasa, MD, MPH**, **Genentech** (Other Financial or Material Support, I receive an honorarium for lectures - it’s a education grant, supported by genetech)**Quidel** (Grant/Research Support, Other Financial or Material Support, Donation of supplies/kits)**Sanofi** (Grant/Research Support, Other Financial or Material Support, HAI/NAI testing) **Natasha B. Halasa, MD, MPH**, Genentech (Individual(s) Involved: Self): I receive an honorarium for lectures - it’s a education grant, supported by genetech, Other Financial or Material Support, Other Financial or Material Support; Sanofi (Individual(s) Involved: Self): Grant/Research Support, Research Grant or Support

